# Acute effects of concurrent muscle power and sport-specific endurance exercises on markers of immunological stress response and measures of muscular fitness in highly trained youth male athletes

**DOI:** 10.1007/s00421-022-05126-8

**Published:** 2023-01-10

**Authors:** Adrian Markov, Jens Bussweiler, Norman Helm, Fabian Arntz, Thomas Steidten, Lars Krohm, Arnau Sacot, Philipp Baumert, Christian Puta, Helmi Chaabene

**Affiliations:** 1 Division of Training and Movement Sciences, Research Focus Cognition Sciences, Faculty of Human Sciences, University of Potsdam, Am Neuen Palais 10, Building. 12, 14469 Potsdam, Germany; 2 Olympic Testing and Training Center Brandenburg, Potsdam, Germany; 3 Department of Sports Medicine and Health Promotion, Friedrich-Schiller-University Jena, 07740 Jena, Germany; 4 University de Girona, Girona, Spain; 5 Exercise Biology Group, Faculty of Sport and Health Sciences, Technical University of Munich, Munich, Germany; 6 Center for Interdisciplinary Prevention of Diseases Related to Professional Activities, Friedrich-Schiller-University Jena, Jena, Germany; 7 High Institute of Sports and Physical Education of Kef, University of Jendouba, 8189 Jendouba, Tunisia

**Keywords:** Concurrent training, Risk of infection, White blood cells, Leucocytosis, Lymphocytosis, Adolescents, Combat sports

## Abstract

**Purpose:**

To examine the acute effects of concurrent muscle power and sport-specific endurance exercises order on immunological stress responses, muscular-fitness, and rating-of-perceived-exertion (RPE) in highly trained youth male judo athletes.

**Methods:**

Twenty male participants randomly performed two concurrent training (CT) sessions; power-endurance and endurance-power. Measures of immune response (e.g., white blood cells), muscular-fitness (i.e., counter-movement-jump [CMJ]), RPE, blood-lactate, and -glucose were taken at different time-point (i.e., pre, mid, post, and post6h).

**Results:**

There were significant time*order interactions for white blood cells, lymphocytes, granulocytes, granulocyte-lymphocyte-ratio, and systemic-inflammation-index. Power-endurance resulted in significantly larger pre-to-post increases in white blood cells and lymphocytes while endurance-power resulted in significantly larger pre-to-post increases in the granulocyte-lymphocyte-ratio and systemic-inflammation-index. Likewise, significantly larger pre-to-post6h white blood cells and granulocytes increases were observed following power-endurance compared to endurance-power. Moreover, there was a significant time*order interaction for blood-glucose and -lactate. Following endurance-power, blood-lactate and -glucose increased from pre-to-mid but not from pre-to-post. Meanwhile, in power-endurance blood-lactate and -glucose increased from pre-to-post but not from pre-to-mid. A significant time*order interaction was observed for CMJ-force with larger pre-to-post decreases in endurance-power compared to power-endurance. Further, CMJ-power showed larger pre-to-mid performance decreases following power-endurance, compared to endurance-power. Regarding RPE, significant time*order interactions were noted with larger pre-to-mid values following endurance-power and larger pre-to-post values following power-endurance.

**Conclusion:**

CT induced acute and delayed order-dependent immune cell count alterations in highly trained youth male judo athletes. In general, power-endurance induced higher acute and delayed immunological stress responses compared to endurance-power. CMJ-force and RPE fluctuated during both CT sessions but went back to baseline 6 h post-exercise.

## Introduction

Acute immunological responses to exercise are multifactorial and marked by systemic alterations of hormone- and immune cell concentration (Gleeson 2007; Kraemer et al. 2020). Compelling evidence suggests that chronic exercise has anti-inflammatory effects (Gleeson et al. 2011). Meanwhile, acute immunological responses to exercise are yet to be discussed (Campbell and Turner 2018a). Nonetheless, it is well-established that exercise is associated with acute leucocytosis in healthy individuals (Walsh et al. 2011). In fact, leucocytosis is one of the most clinically accepted markers of inflammatory response and is often associated with infection (Opdenakker et al. 1998) and exercise-induced stresses (Walsh et al. 2011). The magnitude of leucocytosis is dependent on several training variables such as type of exercise, intensity, and duration (Walsh et al. 2011; Ghanbari-Niaki et al. 2011; Schlagheck et al. 2020; Bessa et al. 2016). In terms of youth athletes, only a few studies investigated acute immunological responses to exercise (Freitas et al. 2016; Moraes et al. 2017; Puta et al. 2018), making our understanding of the matter relatively deficient. Generally, growth and maturation are associated with changes within tissues, organs, body systems, body composition, and physical fitness (DiFiori et al. 2014). Thus, youth athletes are a vulnerable group since high training volumes at early ages may increase not only the risk for infection (Nieman and Wentz 2019; Moreira et al. 2009) but also the likelihood for drop out due to acute or chronic injury (Fabricant et al. 2016; Roberts 2014).

To succeed in competition, elite team and individual athletes seek the development of both, high levels of muscle strength and power as well as cardiorespiratory endurance. In this context, concurrent training (CT) is a commonly applied and effective training approach. It stands for the combination of strength/power and endurance exercises to improve both measures of muscular fitness (e.g., muscle strength and power) and cardiorespiratory endurance (e.g., maximal oxygen consumption). Immunological events in the context of CT received little attention in the literature with the majority of the available studies focused on hormonal responses (Schumann et al. 2013; Schumann et al. 2014; Sparkes 2020; Enright et al. 2018; Inoue et al. 2016). For instance, Schumann and colleagues (Schumann et al. 2013; Schumann et al. 2014) reported that strength followed by endurance exercises induced similar acute peripheral alterations of cortisol and testosterone compared to endurance followed by strength in physically active men. Similar results were reported for professional male soccer players when strength exercises were combined with soccer-specific endurance training or vice versa (Sparkes 2020; Enright et al. 2018). Meanwhile, Inoue et al. (Inoue et al. 2016) investigated the effects of concurrent endurance and strength exercises order on acute inflammatory responses in recreational male weightlifters and found similar acute alterations for interleukin-6, interleukin-10 and tumour-necrosis-factor-alpha immediately post-exercise, regardless of the order. In general, the number of studies in this area is limited and the available ones indicate that the acute immune response to CT may differ from those known after single-mode strength- or endurance training (Donges et al. 2014), yet supportive evidence for youth athletes is missing.

Several factors (e.g., type of exercise, intensity, and duration) are likely to alter the effects of CT (Fyfe et al. 2014; Fyfe and Loenneke 2018; Ihalainen et al. 2017). Particularly, there are indications that the applied exercise order during CT determines the magnitude of the underlying physiological events (Schumann et al. 2013; Coffey and Hawley 2017; Taipale et al. 2014). However, there is hardly any study that examined the effects of CT exercise order on markers of acute immunological stress responses (e.g., While blood cells [WBC], lymphocytes [LYM], granulocytes [GRAN], granulocyte-lymphocyte-ratio [GLR] and the systemic inflammation index [SII]). Of note, the GLR and SII are newly emerging inflammatory markers, mostly used in clinical settings (Buonacera et al. 2022). However, while their use in exercise science is yet limited, they are attracting more attention. This is because the available evidence indicated moderate-to-high correlations of GLR and SII with other well-established inflammatory markers such as the C-reactive-protein and Interleukin-6 (Huang et al. 2018; Islas-Vazquez et al. 2020; Walzik et al. 2021).

Besides, youth elite athletes are yet underrepresented in the literature, although they are commonly exposed to high training loads, which could weaken their immune system, making them prone to injury and infection (DiFiori et al. 2014; Fabricant et al. 2016; Roberts 2014). Therefore, the primary aim of this study was to investigate the effect of CT order (i.e., power-endurance versus endurance-power) on acute (< 15 min) and delayed (≤ 6 h) immunological stress responses (i.e., WBC, LYM, GRAN, GLR, and SII) in highly trained youth male judo athletes. We additionally examined the effects of CT order on measures of muscular fitness (i.e., jump height, force, power) and rating of perceived exertion (RPE). We hypothesized that different exercise orders during CT would cause different acute and delayed alterations in peripheral immune cell counts, measures of muscular fitness, and RPE in highly trained youth male judo athletes (Schumann et al. 2013; Coffey and Hawley 2017; Taipale et al. 2014).

## Methods

### Participants

A total of 24 judo athletes were recruited for the study. To be included, participant had to be free from injuries or any signs of infectious diseases before and throughout the entire experimental period. All participants were enrolled in a national training centre and were members of the federal and/or national judo squat and can therefore be classified as highly trained (McKay et al. 2022). Additionally, they were all actively engaged in national and/or international judo competitions. Their general training routine consisted of two sessions per day, lasting one to two hours each on ≥ 5 days a week. The maturity status of participants was determined using the maturity offset method, which was estimated using the predictive equation of Mirwald et al. (2002) for males. During the wash-out period, four individuals dropped out of the study. Thus, 20 males completed the entire protocol. Details of the included participants at baseline are provided in Table 1. All participants as well as their legal guardians gave written informed consent to participate in the study. The Human Ethics Committee at Potsdam University approved the experimental procedure.

**Table 1:** The characteristics of participants

Characteristics	Value
Number of participants	20
Age (years)	16 ± 1.8
Maturity offset (years)	1.8 ± 1.4
Sitting height (cm)	89.2 ± 3.8
Standing height (cm)	171.2 ± 8
Training age (years)	9.1 ± 1.5
Training volume (hours/week)	17 ± 4
Body mass (kg)	64.7 ± 11.6

### Procedure

**Fig. 1:**
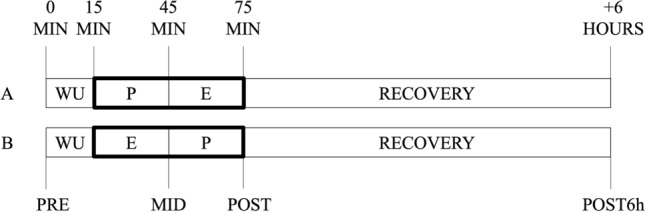
Schematic overview of the protocol. *MIN* minutes, *WU* warm-up, *P* power exercise, *E* sport-specific endurance exercise, *A* power-endurance order, *B* endurance-power order

A schematic overview of the study is shown in Fig. 1. The experiments took place during May and June 2021. All participants visited the training/testing area on four different occasions. During the first visit, all participants were familiarised with all exercises (see “Muscle power and sport-specific endurance exercises” section) and the experimental protocol while during the second visit, the 1-repetition maximum protocol using the leg-press machine was carried out. Thirty-six hours before the third and fourth visits, participants were instructed to avoid any kind of physical activity. Further, all participants were advised to have breakfast on the day of the experiment but to follow their normal filling routine. Due to COVID-19 restrictions, participants were assigned to three groups. The first arrived at 7:00 am, the second at 9:30 am, and the third at 11:30 am. All participants performed a standardised warm-up routine, based on selected exercises of the FIFA 11 + program (FIFA 2022). Then, they were randomly assigned to either the power-endurance or endurance-power order. After the end of the protocols, all participants were informed to follow their daily routine but were instructed not to perform any additional sports activities for the rest of the day. Following a wash-out period of 2 weeks in which all participants followed their normal training routine, the procedure was repeated. More specifically, all participants from the power-endurance order ran endurance-power and vice versa.

### Muscle power and sport-specific endurance exercises

With reference to the recommendations of Faigenbaum et al. (2009) for the progression of resistance training in youth athletes, the power exercise consisted of 4 sets of 8 repetitions using a leg-press machine (SCHNELL, Peutenhausen, Germany) at 30–40% of 1-repetition maximum with 4 min break between sets (total work time including breaks and post measures ⁓ 25 min). This setting is commonly used in practice and aims to improve muscular power. Participants were instructed to perform each repetition as fast as possible. For the sport-specific endurance exercise, the previously validated Special-Judo-Fitness-Test (Sterkowicz et al. 1999) was used. Specifically, four rounds of the Special-Judo-Fitness-Test with three sets (*A* = 15 s; *B* and *C* = 30 s) per round were carried out. The rest in between sets and rounds was 10 s and 4 min, respectively. Briefly, during each round, the evaluated participant throws two partners who are positioned 12 m apart as many times as possible using the *ippon-seoi-nage* technique (Franchini et al. 2011). The total work time including breaks and post measures was ⁓ 25 min. Due to their daily training routine and the previously applied familiarisation sessions, all participants were acquainted with the applied exercises.

### Data collection

All time points of measurement (i.e. pre, mid, post, post6h) are shown in Fig. 1. Capillary blood markers of immune response were obtained from the earlobe (20 µl) at pre, post, and post6h. In this context, we did not measure immune responses between the power and sport-specific endurance protocol (i.e. mid). This was due to the fact, that an additional measure would have extended the timeframe between the strength and sport-specific endurance exercise. WBC, LYM, GRAN, and (blood) platelet were analysed immediately after taking the blood sample by using a haematology analyser system (Medonic M32, Boule Medical AB, Sweden). Medonic M32 uses a WBC-discriminator and operates due to the principle of impedance. Thereby, GRAN includes neutrophils, basophils, and eosinophils. Based on previous literature (Buonacera et al. 2022; Steidten et al. 2021), GLR and the SII were calculated as follows:

$$\mathrm{GLR}=\frac{\mathrm{GRAN}}{\mathrm{LYM}}$$

$$\mathrm{SII}=\mathrm{Platelets}*\frac{\mathrm{GRAN}}{\mathrm{LYM}}$$

Additionally, 10 µl blood were taken from the earlobe at pre, mid, and post, to quantify blood lactate and blood glucose (Biosen S-Line, EKF-Diagnostics, Germany). The RPE using the BORG scale (Williams 2017) and CMJ-height, -force, and -power using a force plate (Leonardo Jumping Platform, Novotec, Germany) were assessed at pre, mid, post, and post6h.

### Statistical analyses

To examine the effects of CT exercise order on the dependent variables (i.e., WBC; LYM; GRAN; GLR; SII; blood lactate and glucose; CMJ-performance [i.e., power; force; height] as well as RPE) a 2 (order: endurance-power and power-endurance) * 3 or 4 (time: Pre, Mid, Post, Post6h) repeated measure analysis of variance was computed. Data were tested and confirmed for normal distribution using the Shapiro–Wilk test. To correct for violations of sphericity, the degrees of freedom were corrected in the normal way, using the Huynh–Feldt (*ε* > 0.75) or Greenhouse–Geisser (*ε* < 0.75) values for ε, as appropriate (Field and Discovering statistics using SPSS 2009). In the case of significant order*time interactions, Bonferroni pairwise comparisons were conducted (Field and Discovering statistics using SPSS 2009; Cohen 1988). Effect size (ES) was interpreted as trivial (ES < 0.20), small (0.2 ≤ ES < 0.50), moderate (0.50 ≤ ES < 0.80) or large (ES ≥ 0.80) (Cohen 1988). The results are presented as mean ± standard deviation (SD). Statistical significance was set at *p* < 0.05. The data were analysed using the Statistical Package for Social Science (SPSS, Chicago, IL, USA, version 29.0).

## Results

Mean values and standard deviations for all measures are displayed in Table 2. There were no baseline differences between the two exercise orders for all measurements.

**Table 2:** Concurrent training order-specific means (± standard deviation) for all outcome measures before (pre), between (mid), immediately after (post), and 6 h after (post6h) the protocols

Variable	Condition	Pre	Mid	Post	Post6h	Order	Time	Time*order interaction, ES
WBC (10^9^/l)	Power-endurance	7.20 ± 2.10		11.80 ± 3.22*	14.25 ± 3.43*	*F*_1, 19_ = 9.89; ***p* = 0.005**	*F*_2, 38_ = 56.34; ***p* < 0.001**	*F*_2, 38_ = 4.25; ***p* = 0.022**, ES = 0.95
	Endurance-power	7.60 ± 2.82		10.10 ± 2.53*	11.86 ± 2.30*			
GRAN (10^9^/l)	Power-endurance	3.75 ± 1.25		5.99 ± 1.79	9.77 ± 2.99*	*F*_1, 19_ = 0.23; *p* = 0.638	*F*_2, 38_ = 61.87; ***p* < 0.001**	*F*_1.94, 36.80_ = 6.73; ***p* = 0.004**, ES = 1.19
	Endurance-power	4.11 ± 2.30		6.90 ± 2.57	7.98 ± 2.00*			
LYM (10^9^/l)	Power-endurance	2.97 ± 1.08		4.99 ± 2.05*	3.55 ± 1.10	*F*_1, 19_ = 23.63; ***p* < 0.001**	*F*_1.80, 34.16_ = 5.54; ***p* = 0.010**	*F*_1.68, 32.00_ = 16.90; ***p* < 0.001**, ES = 1.89
	Endurance-power	2.96 ± 0.93		2.59 ± 0.89*	3.11 ± 1.09			
GLR (10^9^/l)	Power-endurance	1.35 ± 0.50		1.36 ± 0.56*	2.91 ± 1.17	*F*_1, 19_ = 5.48; ***p* = 0.030**	*F*_1.86, 35.36_ = 31.34; ***p* < 0.001**	*F*_2, 38_ = 11.39; ***p* < 0.001**, ES = 1.55
	Endurance-power	1.44 ± 0.74		2.97 ± 1.63*	2.94 ± 1.42			
SII (10^9^/l)	Power-endurance	203 ± 75		209 ± 110*	427 ± 214	*F*_1, 19_ = 6.06; ***p* = 0.024**	*F*_2,_ 38 = 17.56; ***p* < 0.001**	*F*_2, 38_ = 8.63; ***p* < 0.001**, ES = 1.35
	Endurance-power	219 ± 116		456 ± 290*	444 ± 252			
LA (mmol/l)	Power-endurance	0.99 ± 0.29	1.50 ± 0.35*	8.06 ± 2.89*		*F*_1, 19_ = 22.84; ***p* < 0.001**	*F*_1.48, 28.17_ = 108.65; ***p* < 0.001**	*F*_1.13, 21.46_ = 136.27; ***p* < 0.001**, ES = 5.37
	Endurance-power	0.97 ± 0.20	9.19 ± 3.02*	2.72 ± 0.94*				
GLU (mmol/l)	Power-endurance	4.74 ± 0.88	4.54 ± 0.63*	5.54 ± 0.93*		*F*_1, 19_ = 2.13; *p* = 0.160	*F*_1.29, 24.43_ = 2.34; *p* = 0.133	*F*_2, 38_ = 11.60; ***p* < 0.001**, ES = 1.56
	Endurance-power	4.92 ± 1.16	5.82 ± 1.18*	4.57 ± 0.71*				
CMJ-H (cm)	Power-endurance	41.49 ± 6.32	40.68 ± 6.02	40.10 ± 5.46	42.31 ± 6.74	*F*_1, 19_ = 1.26; *p* = 0.276	*F*_3, 57_ = 3.10; ***p* = 0.034**	*F*_1.84, 35.01_ = 0.50; *p* = 0.595, ES = 0.33
	Endurance-power	40.78 ± 5.80	40.73 ± 6.66	39.12 ± 7.68	40.87 ± 6.29			
CMJ-P (W)	Power-endurance	3.15 ± 0.90	3.12 ± 0.89*	3.17 ± 0.86	3.21 ± 0.93	*F*_1, 19_ = 1.09; *p* = 0.310	*F*_3, 57_ = 2.50; *p* = 0.068	*F*_2.67, 50.68_ = 3.80; ***p* = 0.019**, ES = 0.90
	Endurance-power	3.15 ± 0.93	3.23 ± 0.98*	3.00 ± 0.94	3.16 ± 0.87			
CMJ-F (N)	Power-endurance	1.56 ± 0.41	1.53 ± 0.43	1.57 ± 0.40*	1.54 ± 0.40	*F*_1, 19_ = 0.34; *p* = 0.567	*F*_3, 57_ = 0.76; *p* = 0.521	*F*_2.04, 38.84_ = 3.34; ***p* = 0.045**, ES = 0.84
	Endurance-power	1.59 ± 0.43	1.57 ± 0.43	1.51 ± 0.39*	1.56 ± 0.39			
RPE (score)	Power-endurance	7 ± 1	9 ± 1*	18 ± 2*	7 ± 1	*F*_1, 19_ = 4.02; *p* = 0.059	*F*_2.17, 41.15_ = 274.94; ***p* < 0.001**	*F*_2.73, 51.82_ = 285.51; ***p* < 0.001**, ES = 7.78
	Endurance-power	7 ± 1	18 ± 1*	11 ± 2*	8 ± 2			

Values in bold highlight significant effects

*WBC* white blood cells, *LYM* lymphocytes, *GRAN* granulocytes, *GLR* granulocyte-lymphocyte-ratio, *SII* systemic inflammation index, *LA* blood lactate, *GLU* glucose, *CMJ-H* countermovement jump height, *CMJ-P* countermovement jump power, *CMJ-F* countermovement jump force, *RPE* rate of perceived exertion, *PRE* before the protocol, *MID* in between the protocol, *POST* immediately after the protocol, *POST6h* six hours after the protocol, *ES* Effect size

*Significant time × order interaction effect

### Blood markers of immune response

**Fig. 2:**
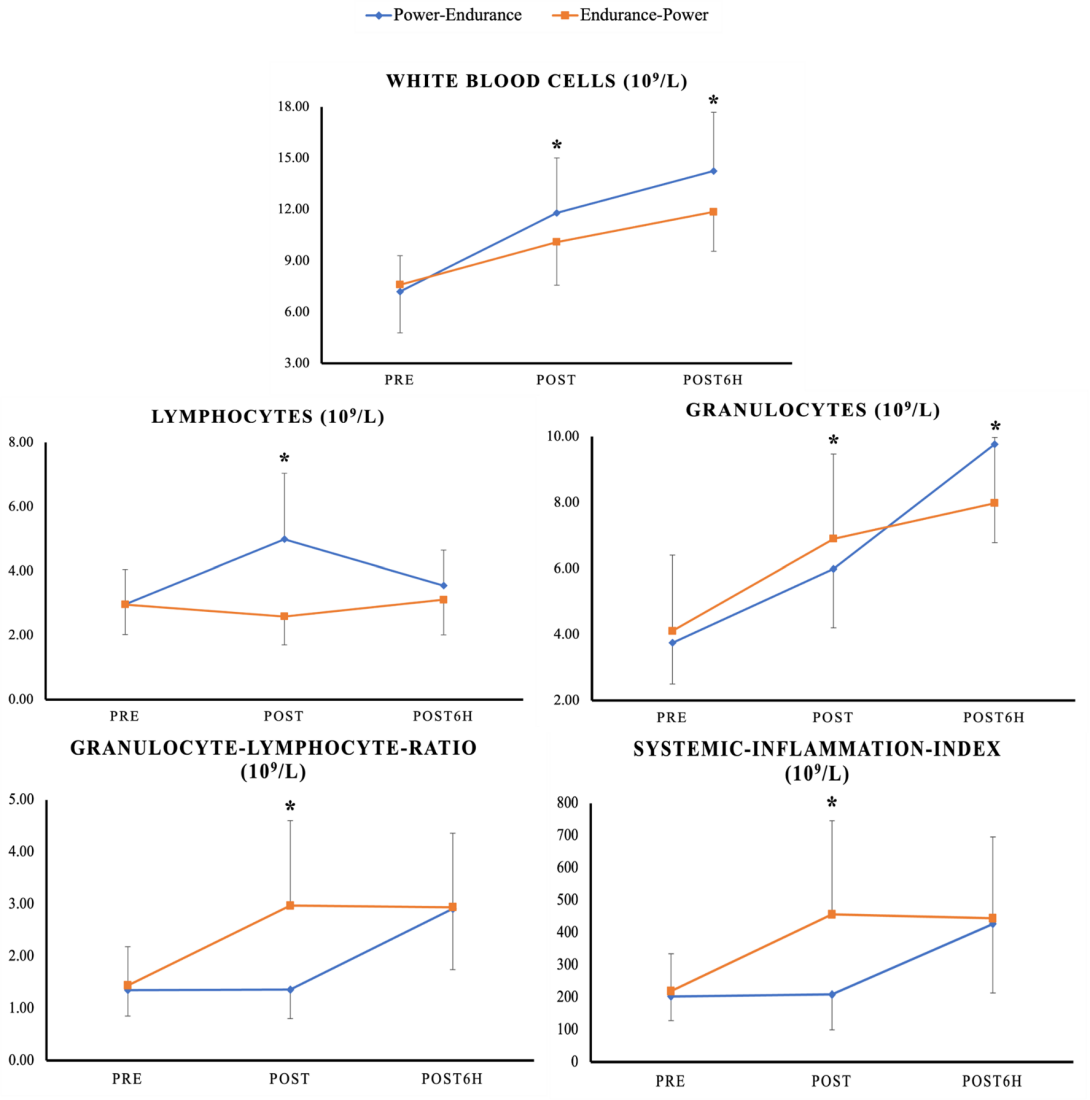
Means and standard deviation for all immunological blood markers measured at pre, post, and 6 h post for the power-endurance (blue coloured line) and endurance-power order (orange coloured line). The graph highlights that concurrent training induced order-dependent immune cell count alterations in healthy youth male judo athletes. From an acute (≤ 15 min) perspective, significant differences between the two exercise orders in white blood cells, lymphocytes, granulocyte-lymphocyte-ratio, and the systemic inflammation index were observed. From a delayed (≤ 6 h) perspective, there were significant differences in white blood cells and granulocytes. *Stands for significant time*order interaction effect

Results showed significant time*order interactions for WBC (*p* = 0.022), LYM (*p* < 0.001), GRAN (*p* = 0.004), GLR and SII (*p* < 0.001, respectively). More specifically, power-endurance resulted in significantly larger pre-to-post (∆ + 64%; ES = 2.20) and pre-to-post6h (∆ + 98%; ES = 3.40) increases for WBC compared to endurance-power (∆ + 33%; ES = 0.90 and ∆ + 55%; ES = 1.50, respectively). For LYM, significantly larger pre-to-post increases (∆ + 68%; ES = 1.87) in power-endurance compared to endurance-power (∆ − 13%, ES = 0.40) were noted. In terms of GRAN, results indicated significantly larger pre-to-post6h elevations (∆ + 160%; ES = 4.82) for power-endurance compared to endurance-power (∆ + 94%; ES = 1.68). Regarding GLR and SII, findings indicated significantly larger pre-to-post increases for endurance-power (∆ + 106%; ES = 2.07 and ∆ + 109%; ES = 2.05, respectively), compared to power-endurance (∆ + 1%; ES = 0.02 and ∆ + 3%; ES = 0.08, respectively). A graphical representation of these results can be found in Fig. 2.

### Metabolic response

**Fig. 3:**
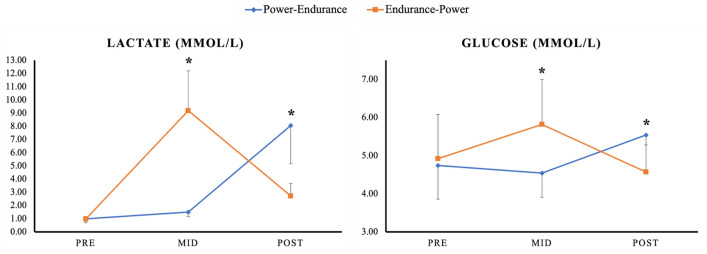
Means and standard deviation for all metabolic values collected at pre, mid, post, and 6 h post for the power-endurance (i.e., blue coloured line) and endurance-power order (i.e., orange coloured line). The graph highlights significant time*order interaction effects with significantly larger pre-to-mid increases in blood glucose and lactate following the endurance exercise (as part of the endurance-power order) compared to significantly larger pre-to-post increases in blood glucose and lactate following the endurance exercise (as part of the power-endurance order). *Stands for significant time*order interaction effect

Our findings indicated significant time*order interactions for blood glucose and lactate (*p* < 0.001, respectively). Results showed significantly larger pre-to-mid increases in blood glucose and lactate following the endurance exercise (as part of the endurance-power order, ∆ + 18%; ES = 0.78 and ∆ + 947%; ES = 40.50, respectively) compared to the power exercise (as part of the power-endurance order, ∆ − 4%; ES = 0.23 and ∆ + 52%; ES = 1.72, respectively). From pre-to-post, changes in blood glucose and lactate were significantly larger for the power-endurance order (∆ + 17%; ES = 0.91 and ∆ + 814%; ES = 24.38, respectively), compared to the endurance-power order (∆ − 7%; ES = 0.30 and ∆ + 280%; ES = 8.75, respectively). A graphical representation of these results can be found in Fig. 3.

### Physical performance and subjective perception of effort

**Fig. 4:**
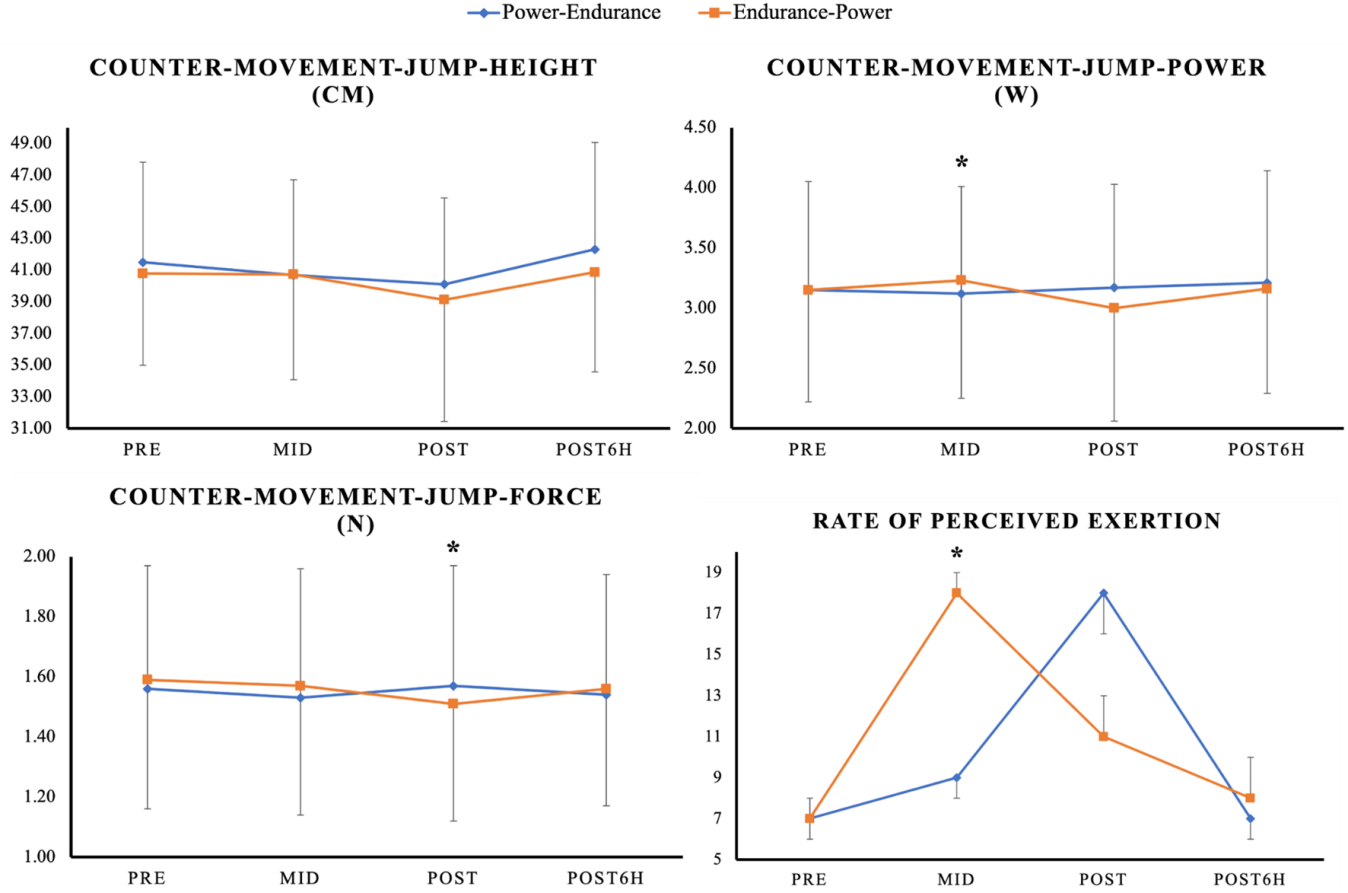
Means and standard deviation for all physical performance and perceived exertion values collected at pre, mid, post, and 6 h post for the power-endurance (i.e., blue coloured line) and endurance-power order (i.e., orange coloured line). The graph highlights significant time*order interactions for CMJ-performance and rate of perceived exertion. CMJ-force showed significantly larger pre-to-post performance decreases within the endurance-power order while CMJ-power showed larger pre-to-mid performance decreases following the power-endurance order. Rate of perceived exertion was significantly higher following the endurance exercise (as part of the endurance-power order. *Stands for significant time*order interaction effect

For physical performance, a significant time*order interaction was observed for CMJ-force (*p* = 0.045) with significantly larger pre-to-post performance decreases for the endurance-power order (∆ − 5%; ES = 0.19), compared to the power-endurance order (∆ + 1%; ES = 0.02). Further, there was a significant time*order interaction for CMJ-power (*p* = 0.019) with larger pre-to-mid performance decreases for the power-endurance order (∆ − 1%; ES = 0.03), compared to the endurance-power order (∆ + 3%; ES = 0.09). Regarding RPE, there was a significant time*order interaction (*p* < 0.001) showing larger pre-to-mid values following the endurance exercise (as part of the endurance-power order, ∆ + 157%; ES = 11.00), compared to the power exercise (as part of the power-endurance order, ∆ + 29%; ES = 2.00). Additionally, significantly larger pre-to-post RPE values were observed for power-endurance order (∆ + 157%; ES = 11.00), compared to endurance-power (∆ + 57%; ES = 4.00). A graphical representation of these results can be found in Fig. 4.

## Discussion

The main findings of this study indicated that CT induced acute (≤ 15 min) and delayed (≤ 6 h), order-dependent immune cell count alterations in healthy youth male judo athletes. In general, the power-endurance order induced higher increases in WBC compared to the endurance-power order. Further, we found order-dependent fluctuations in CMJ-force with larger performance decreases after endurance-power compared to power-endurance while CMJ-power significantly increased from pre-to-mid following the endurance-power order. For RPE, findings indicated larger values from pre-to-post following power-endurance while endurance-power induced larger values from pre-to-mid.

This is the first study investigating the acute effects of CT across two different exercise orders on immunological stress responses in youth male judo athletes. Consequently, comparative literature is sparse. In general, physical exercise that involves repetitive muscle contraction acutely stimulates the activity of the central nervous system and causes systemic substrate metabolism (Valencia-Sánchez et al. 2019). This would alter immune cell activity/function and further, stimulate the release of glucocorticoid hormones (e.g., cortisol), catecholamines (e.g., adrenaline), cytokines (e.g., Interleukin-6), and WBC into peripheral circulation (Valencia-Sánchez et al. 2019; Rosa-Neto et al. 2022). Thereby, the increase in WBC seems to be due to demargination from the vessel walls and to cell releases from organic storage (e.g., liver, lung), as well as the thymus gland, bone marrow lymph nodes, and skeletal muscle (Simpson et al. 2015). The magnitude and the time course of exercise-induced leucocytosis and thus, any alteration within circulating immune cell counts is dependent on several factors, such as age, duration and intensity and/or the type of the applied exercise (Walsh et al. 2011; Schlagheck et al. 2020; Natale et al. 2003). To the best of our knowledge, acute leucocytosis in responses to power-based exercises is yet to be investigated. Previous studies reported acute leucocytosis after strength (Ihalainen et al. 2014; Nieman et al. 1995), endurance (Nielsen et al. 1996; Shek et al. 1995; Vider et al. 2001; Wahl et al. 2020), and concurrent strength and endurance exercises (Bessa et al. 2016), in trained (Bessa et al. 2016; Nielsen et al. 1996; Vider et al. 2001; Wahl et al. 2020) and recreationally active (Ihalainen et al. 2014; Nieman et al. 1995; Shek et al. 1995) adults. Although a direct comparison to most of the forenamed studies is difficult, our findings generally corroborate with the literature since both exercise orders induced acute (≤ 15 min) and delayed (≤ 6 h) immune cell count alterations (i.e. WBC, LYM, GRAN) in youth highly trained athletes. However, based on our results it seems that the magnitude of acute and delayed immune cell count alterations to CT is exercise order-dependent with particularly higher increases in WBC following the power-endurance order compared to the endurance-power order (Fig. 2).

Generally, LYM make up to 40% of the total WBC count in the bloodstream (Kverneland et al. 2016). LYM are made of lymphoid stem cells within the bone marrow and act as a crucial part of the adaptive immune system. It is accepted that lymphocytosis originates during and immediately after exercise, mainly due to an adrenergic stimulation forcing LYM to detach from vessel walls into circulation (Campbell and Turner 2018b). However, LYM fall back and could reach values even below baseline shortly after exercise (≤ 30 min). Several attempts have previously been made to explain the rapid fall-back of LYM (Vider et al. 2001; Nieman 2000; Kakanis et al. 2010; Shinkai et al. 1996). More specifically, recent literature indicates that LYM shift to peripheral tissue to obtain an increased state of immune surveillance (Campbell and Turner 2018b; Krüger et al. 2008; Kruger and Mooren 2007). It is widely accepted that the magnitude of lymphocytosis and lymphocytopenia is proportional to the applied duration and intensity of exercise (Walsh et al. 2011; Campbell and Turner 2018b). With respect to the current study, LYM solely increased in the power-endurance order, from pre-to-post but then decreased from post towards post6h. For the endurance-power order, LYM decreased from pre-to-post, before slightly increasing towards post6h. Therefore, we would report an acute but not delayed LYM difference between the two exercise orders. In fact, acute lymphocytosis was expected after both exercise orders. This is because the overall exercise load was equal between both exercise orders. In this context, we need to note that we did not measure cell kinetics between the power and sport-specific endurance protocol (i.e., mid). As such, it could be assumed that we have missed the time point in which LYM were elevated within the endurance-power order.

GRAN represents the main component of total WBC (up to 60%) in healthy young adults (Kverneland et al. 2016). GRAN mature within bone marrow (Walsh et al. 2011) and rise during and after exercise several folds (Schlagheck et al. 2020; Natale et al. 2003; Nieman et al. 1995; Nieman et al. 1989). Unlike LYM, GRAN are made of myeloid stem cells and are responsible for the innate immune response. GRAN partly circulates throughout the peripheral system while adhering to the endothelial surface. Exercise-induced haemodynamics (e.g. increased blood flow, shear stress) trigger GRAN to demarginate from the blood vessel walls, which acutely increases the number of circulating cells (Simpson et al. 2015). The general trend of post-exercise GRAN kinetics was previously shown as a continuous increase, which starts during exercise but lasts up to six hours, depending on the applied exercise (Nieman et al. 1989; Ramel et al. 2003; Gabriel et al. 1992; Mayhew et al. 2005). Therefore, our results corroborate with the existing literature as GRAN gradually increased regardless of the applied exercise order. Although, results indicated a markedly higher delayed (i.e., pre-to-post6h) increase in GRAN following power-endurance (∆161%) compared to endurance-power (∆94%). However, the significant difference between the two exercise orders in the delayed cell count was unexpected. In this context, cortisol and catecholamines play a key role in activating the immune system and particularly, GRAN release from the bone marrow (Peake et al. 2017). Interestingly, it was previously suggested that the release of cortisol and catecholamines, is intensity-dependent (Hill et al. 2008; Kindermann et al. 1982). Although we did not measure hormone levels, it was interesting to find significant time*order interactions for RPE from pre-to-mid, indicating that the sport-specific endurance exercise imposed a higher physiological stress compared to the muscle power exercise. Consequently, it seems that the sport-specific endurance exercise was the main driver of the observed immune responses but that, in the endurance-power order, the applied power exercise may have partly attenuated the delayed GRAN increase. Based on our findings we would argue that the innate (i.e. GRAN) and adaptive (i.e., LYM) immune systems displayed a distinct time-dependent activity. However, this is a preliminary interpretation that should be confirmed by future studies.

The application of GLR and SII within the area of exercise science is yet limited. Meanwhile, from a clinical perspective, they are well-established markers of diseases (Buonacera et al. 2022) and death (Fois et al. 2020; Moisa et al. 2021). For example, Fois et al. (2020) reported that the SII on admission independently predicts in-hospital mortality in COVID-19 patients. It is worth noting though, that earlier studies demonstrated moderate-to-high correlations of GLR and SII with other well-established inflammatory markers such as the C-reactive-protein and Interleukin-6 (Huang et al. 2018; Islas-Vazquez et al. 2020; Zhu et al. 2020). A recent review by Walzik and colleagues (2021) suggested the application of GLR and SII as feasible tools to assess exercise-induced strain and indicators of recovery processes. Most of the available studies which measured GLR and SII in an exercise setting assessed the effects of endurance exercises on GLR and/or SII. For instance, Wahl et al. (2020) compared different recovery strategies following either high-intensity or sprint interval cycling and its effects on immune cell kinetics in male cyclists/triathletes aged 25 years. The authors reported that irrespective of the applied exercise, GLR and SII increased significantly up to 3 h post-exercise. In another study, Bessa et al. (2016) examined the effects of CT on biomarkers of injury and inflammation in elite cyclists aged 28 years. The authors reported a significant increase in GLR, lasting for 3 h while dropping significantly below baseline 12 h post-exercise. With respect to our results, there were significant time*order interaction effects for GLR and SII. Following endurance-power, GLR and SII increased one-fold immediately post-exercise and stayed elevated after 6 h. Meanwhile, in power-endurance, both parameters did not change from pre-to-post but rise towards post6h to a similar level compared to endurance-power. In general, GLR (SII)-values rise when GRAN (and platelets) are high while LYM are low (Walzik et al. 2021). Our results seem to be reasonable considering the elevated LYM following the power-endurance order but not endurance-power.

A secondary aim of this study was to examine the effect of CT order on CMJ performance and RPE. Our results showed a significantly larger pre-to-post decrease in CMJ-force following endurance-power and larger pre-to-mid performance decreases in CMJ-power for the power-endurance order, compared to the endurance-power order. Additionally, significantly larger pre-to-post RPE values were observed for the power-endurance order compared to endurance-power. However, from pre-to-post6h RPE and CMJ-force and power went back towards baseline. Based on the literature, it is highly recommended to report aspects of internal and external load to give the most accurate feedback about the experienced effort (Balsalobre-Fernández et al. 2014; Halson 2014; Cardinale and Stone 2006; García-Pinillos et al. 2021; Impellizzeri et al. 2019; McLaren et al. 2018). Regarding CT, the study of Bessa et al. (2016) showed an inverse relationship between GLR and upper-body muscle strength in elite male cyclists. More specifically, the authors revealed that GLR significantly increased 3 h post-exercise while muscle strength significantly decreased. However, both values returned to baseline levels after 48 h. With reference to Fig. 4, CMJ-force significantly decreased from pre-to-post and CMJ-power significantly increased from pre-to-mid following the endurance-power order. CMJ-height, on the other hand, did not exhibit a clear fluctuation throughout the two CT orders. Meanwhile, subjective RPE showed changes throughout the protocols. Specifically, compared to the immune cell kinetics which showed elevated cell counts at post and post6h, RPE went up throughout the protocols but went back to baseline 6 h post-exercise. This would indicate that both exercise orders were in fact, physiologically demanding. Of note, our findings pointed towards a disparity between immunological, perceived as well as physical responses following both CT orders. More specifically, while the immunological responses indicated the need for an extended rest to regain immunological homeostasis (Fig. 2), perceived and physical responses showed that athletes seemed to be ready for another session, 6 h post-CT (Fig. 4). Therefore, practitioners should be aware that measures of internal load may differ from those of external load. Nevertheless, these findings should be confirmed by future studies.

### Limitations and future research perspectives

This study has some limitations that warrant discussion. First, cell kinetics were measured at pre, post, and post6h but not in between the power and sport-specific endurance protocol (i.e., mid) or beyond the 6 h post-effort (e.g., 12 h or 24 h post). Additional measures at mid and/or post 12 h and 24 h would have helped provide a better overview of the delayed effects of CT order on the selected markers of the immunological stress response. However, because of the athletes’ congested training schedule besides the COVID-19 restrictions, it was not possible to collect further blood samples beyond 6 h post. Also, blood samples required immediate analyses. This would have led to experimental delays, due to the short time frame between the power- and endurance exercises. Second, contrasting endurance and/or power training alone with endurance-power and power-endurance would have led to a more comprehensive comparison. These particular shortcomings should be considered in future investigations. Third, we applied one power exercise, only. Indeed, to be in line with real-world scenarios, including several exercises may be more suitable when investigating combined power and endurance sessions. Nonetheless, the applied procedure is common practice and was agreed upon in consultation with the coaching staff. Of note, studies that investigated the effects of power exercises and CT on WBC alterations are yet missing. Accordingly, any comparison made to other studies should be interpreted with caution. Also, we considered male athletes only, making any conclusions regarding female athletes difficult. Lastly, it needs to be stressed that we have relied on indirect markers of inflammation (i.e., GLR and SII). There is evidence that both GLR and SII display moderate-to-high associations with other well-established inflammatory markers such as the C-reactive-protein and Interleukin-6 (Huang et al. 2018; Islas-Vazquez et al. 2020; Zhu et al. 2020). Nevertheless, future studies should favour more prominent markers (e.g., cytokines) to provide a more distinct inflammatory status.

### Conclusion

The main findings of this study indicated that concurrent power and sport-specific endurance exercises induced acute (≤ 15 min) and delayed (≤ 6 h), order-dependent immune cell count alterations in healthy youth male judo athletes. Specifically, results showed that immunological stress responses were generally higher after power-endurance compared to endurance-power. However, the mechanisms behind this phenomenon remain speculative. The second finding of this study was that measures of muscular fitness (e.g., CMJ-force) and RPE fluctuated throughout the CT protocol but went back to baseline values 6 h post-exercise. Lastly, it is worth noting that our findings pointed towards a disparity between immunological, perceived as well as physical responses following both CT orders.

## Data Availability

All data are freely available on repositories of the Open Science Framework (https://osf.io/snqkb/).

## Abbreviations

CT: Concurrent training
CMJ: Countermovement jump
GRAN: Granulocytes
GLR: Granulocyte-lymphocyte-ratio
LYM: Lymphocytes
RPE: Rate of perceived exertion
SII: Systemic inflammation index
WBC: White blood cells
